# Unscreened Polaron Ordering Against High Electron Density on Mechanically Cleaved Surfaces of WO_3_ (001) Films

**DOI:** 10.1002/smll.202504162

**Published:** 2025-09-01

**Authors:** Gyubin Lee, Jeongdae Seo, Wooin Yang, Ji‐Ho Mun, Minho Kang, Ho‐Hyun Nahm, Yong‐Hyun Kim, Tae‐Hwan Kim, Chan‐Ho Yang, Yeongkwan Kim

**Affiliations:** ^1^ Department of Physics Korea Advanced Institute of Science and Technology Daejeon 34141 South Korea; ^2^ Department of Physics Pohang University of Science and Technology Pohang 37673 South Korea; ^3^ School of Physics, Institute of Science Suranaree University of Technology Nakhon Ratchasima 30000 Thailand

**Keywords:** 2DEG, perovskites, small polarons

## Abstract

The 2D electron gas (2DEG) is a successful testbed for investigating both small and large polarons, governed by enhanced fundamental couplings through dimensional confinement. However, accessible polaronic states are typically limited to low carrier densities, as polarons collapse due to enforced electronic screening at high densities. This has limited exploration into polaron interactions, including bipolaron formation, inter‐polaron correlations, and broader collective polaron behaviors. Here, an unscreened polaronic state resilient to high electron densities, formed on the mechanically cleaved surface of a WO_3_ (001) film, is reported. Using angle‐resolved photoemission spectroscopy (ARPES) and atomic‐scale scanning tunneling microscopy/spectroscopy (STM/STS), characteristic signatures of polaron formation are observed in the spectra of a surface‐confined metallic 2DEG with an electron density of 2.6 × 10^14^ cm^−2^, generated by a lack of oxygen ions at the surface. Notably, these vacancies adhere to the ***c*** (2 × 2) surface reconstruction, which is stabilized by the cooperation of excess electrons and surrounding lattice distortion, i.e., the formation of a polaron and its ordering, as revealed by the first‐principles calculations. It is anticipated that this ordered polaron phase at high electron densities will pave new pathways for investigating exotic phases related to correlated polaron states or collective polaron movements, like bipolaron superconductivity.

## Graphical abstract

Unscreened high‐density polarons are discovered in a metallic WO_3_ (001) 2DEG, stabilized by surface oxygen vacancies and c(2 × 2) reconstruction. ARPES and STM/STS reveal polaron formation and ordering, supported by first‐principles calculations. This work opens new avenues for exploring collective polaron phenomena, including bipolaron interactions and polaron‐driven exotic phases.

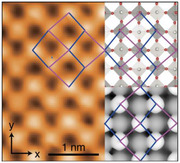

## 1 Introduction

Interactions among polarons^[^ ^1^, ^2^, ^3^, ^4^, ^5^, ^6^ ^]^‐quasiparticles comprising an electron coupled with a distortion in the underlying lattice^[^ ^7^, ^8^, ^9^ ^]^‐are facilitated by strong electron‐phonon coupling and elastic compliance of the host materials. Starting from the simple example of bipolarons,^[^ ^10^, ^11^ ^]^ which are composed of bound pairs of polarons, correlated and/or collective states of polarons can be considered as the origin of nontrivial electronic properties or phenomena.^[^ ^10^, ^11^, ^12^, ^13^, ^14^, ^15^, ^16^, ^17^, ^18^, ^19^ ^]^ Notably, the condensation of bipolarons could transition into superconductivity, namely, the bipolaron superconductivity.^[^ ^10^, ^11^, ^12^, ^13^, ^14^ ^]^ This has been explored as a potential mechanism behind superconductivity in 2D electron gas (2DEG)^[^ ^15^ ^]^ and even high‐*T*_C_ cuprate superconductors.^[^ ^16^, ^17^, ^18^ ^]^ Furthermore, polaron ordering and its subsequent melting are considered crucial for phenomena such as colossal magnetoresistance^[^ ^19^ ^]^ and the pseudogap opening in manganites.^[^ ^20^ ^]^

Recently, low‐dimensional electron systems have been marked as successful platforms for investigating polaronic states, by virtue of enhanced electron‐phonon coupling under dimensional confinement.^[^ ^15^, ^21^, ^22^, ^23^ ^]^ The formation of small (Holstein) polarons has been reported in transition metal dichalcogenide systems,^[^ ^24^ ^]^ and evidence of large (Fröhlich) polaron formation has been captured in the photoemission spectra of 2D electron gas/liquid (2DEG/L) on the surfaces of SrTiO_3_^[^ ^15^, ^21^ ^]^ and anatase TiO_2_.^[^ ^25^, ^26^ ^]^ Despite these successes, experimental investigations into possible interactions of polarons or correlations among them have been limited. As demonstrated in a hallmark experiment on polarons at the SrTiO_3_ surface,^[^ ^15^, ^21^ ^]^ the polaron state collapses at high carrier densities due to increased electronic screening before reaching sufficient density for ordering or interaction. Therefore, to explore stable polaronic states at high electron densities, systems with stronger electron‐phonon interactions are required.

**Figure 1:**
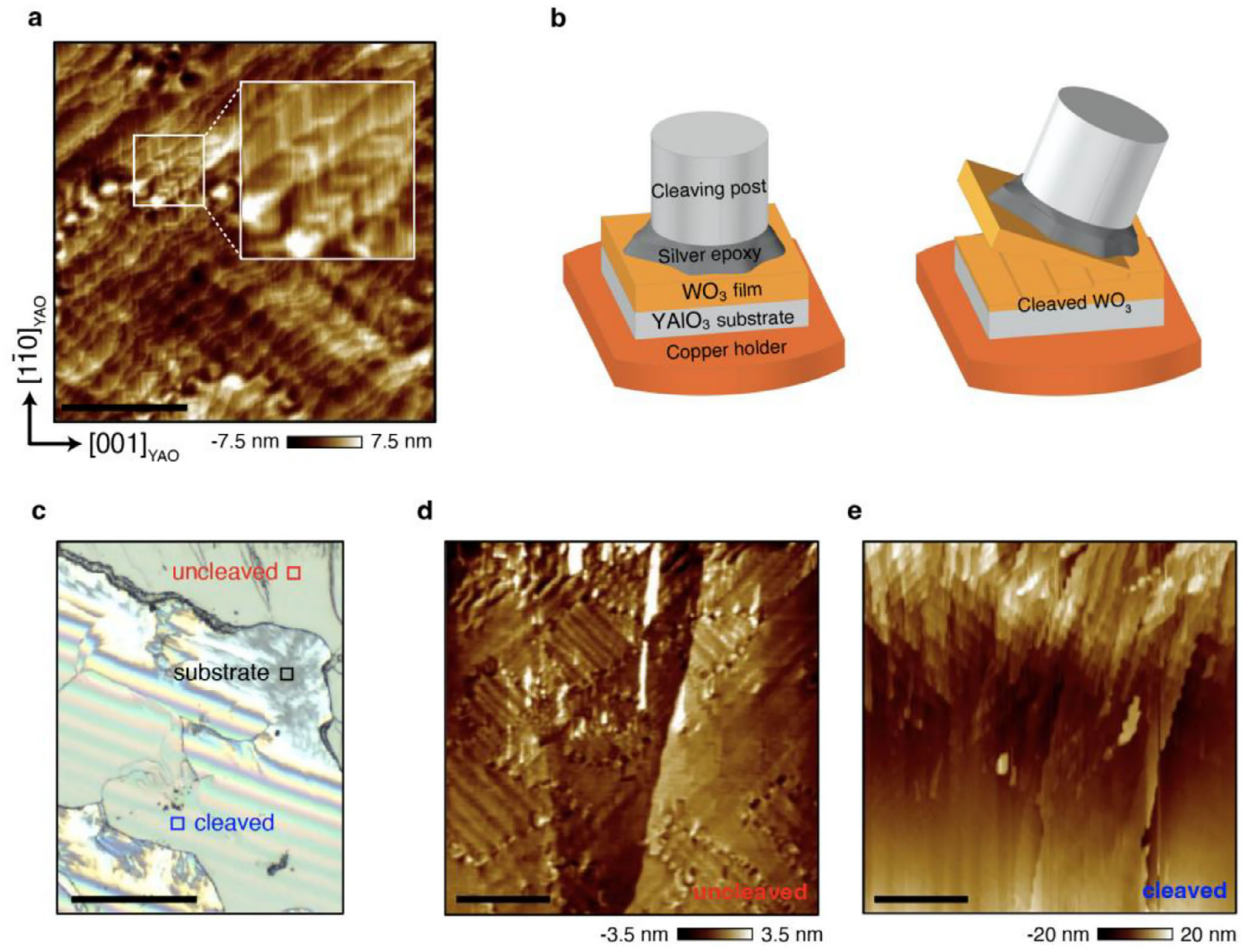
Mechanical cleaving and material characterization on WO_3_ film. a) AFM image of the WO_3_ film surface showing the ferroelastic twin structure with orientations of the YAlO_3_ substrate (scale bar 1 µm). b) The mechanical cleaving process of WO_3_ film. c) Optical microscope image after the cleaving process (scale bar 200 µm). d,e) Surface morphologies of the uncleaved d) and cleaved regions e) (scale bar 5 µm).

Ferroelastic WO_3_, a representative system for polaron and bipolaron formation, is an ideal candidate.^[^ ^14^ ^]^ WO_3_ undergoes a series of structural transitions, indicating the nearly degenerate nature of multiple structural phases.^[^ ^27^ ^]^ The A‐site vacant perovskite ABO_3_ structure of WO_3_ tends to distort or tilt the WO_6_ octahedrons in response to additional stimuli, such as alkali metal (electron) doping. Even in pure WO_3_, similar structural deformations can be induced by oxygen deficiency or twin walls, where the electrical conductivity becomes finite. Furthermore, in the symmetry‐lowered phase, the system exhibits additional degeneracy of four kinds of principal ferroelastic domain states (A_1_, A_2_, B_1_, and B_2_), which form fine‐domains and macro‐domains^[^ ^29^ ^]^ as shown in **Figure** **1**a. This natural instability of the crystal, along with the associated changes in electronic structure of the partially filled 5*d* orbital of WO_3_, is expected to give rise to strong electron‐phonon interactions.

In this work, we report the ordered polaronic state on the mechanically cleaved surface of a WO_3_ thin film grown on a YAlO_3_ substrate, investigated using surface‐sensitive tools such as atomic force microscopy (AFM), scanning tunneling microscopy/spectroscopy (STM/S), and angle‐resolved photoemission electron spectroscopy (ARPES), along with theoretical identification based on density functional theory (DFT) calculations. AFM and STM reveal that a macroscopically flat and atomically clean surface was secured by mechanical cleaving under ultra‐high vacuum (UHV) conditions (see Figure S1, Supporting Information for details), with two oxygen vacancies ordered in a *c*(2 × 2) surface structure. ARPES and STS measurements consistently reveal the metallic state confined to the surface with a high carrier density of ≈2.6 × 10^14^ cm^−2^. Interestingly, the polaronic behavior of this metallic state is evidenced in the spectra from both measurements, paralleling the expected strong electron‐phonon coupling in the present system. Our DFT analysis reveals that polaron ordering, including octahedral rotation at the surface, is the ground state of the system.

## 2 Results and Discussion

### 2.1 Mechanical Cleaving and Material Characterization on WO_3_ Film

First, we deposited 2 µm‐thick WO_3_ films on (110)_o_ YAlO_3_ substrate using pulsed laser deposition (see the Method part and the reference^[^ ^30^ ^]^ for details). The WO_3_ films exhibit a monoclinic phase (space group P2_1_/n, Hermann‐Mauguin notation) with pseudocubic lattice parameters of *a* = 3.66 Å, *b* = 3.76 Å, *c* = 3.85 Å, and *β* = 89.2° (Refs.[30, 31]). Since the pseudocubic lattice parameters (3.71 Å on average) of the orthorhombic substrate lie between the *a* and *b* of WO_3_, two differently oriented monoclinic ferroelastic domains (called macro‐domains) are arranged alternately such that either the *a*‐ or *b*‐axis of WO_3_ is parallel to an in‐plane axis of the substrate, and then cooperative mosaic rotations occur within each macro‐domain so that the *c* axis is perpendicular to the substrate, forming fine‐domains. The unique hierarchical twin structure^[^ ^30^ ^]^ results in a herringbone‐shaped surface morphological feature that appears superimposed on the step terrace structure (Figure 1a).

To evaluate the quality of the cleaved surface of the WO_3_ film after the conventional process illustrated in Figure 1b, we used optical microscopy. The iridescent reflection regions indicate the presence of nanoscale fringes, which result from a coherent and continuous change in the thickness (Figure 1c). Therefore, we attributed these iridescent reflection regions to the cleaved areas of the sample, while the other regions were considered uncleaved. Upon closer inspection with AFM (Figure 1d,e), it appeared that a part of WO_3_ had been ripped off in the cleaved region. The faint macro‐domain patterns observed in this region suggest that the twin structure characteristics are still present. We subsequently performed ARPES and STM measurements on this cleaved region.

### 2.2 STM/STS Analysis of a Cleaved Single‐Crystalline WO_3_ Film

**Figure 2:**
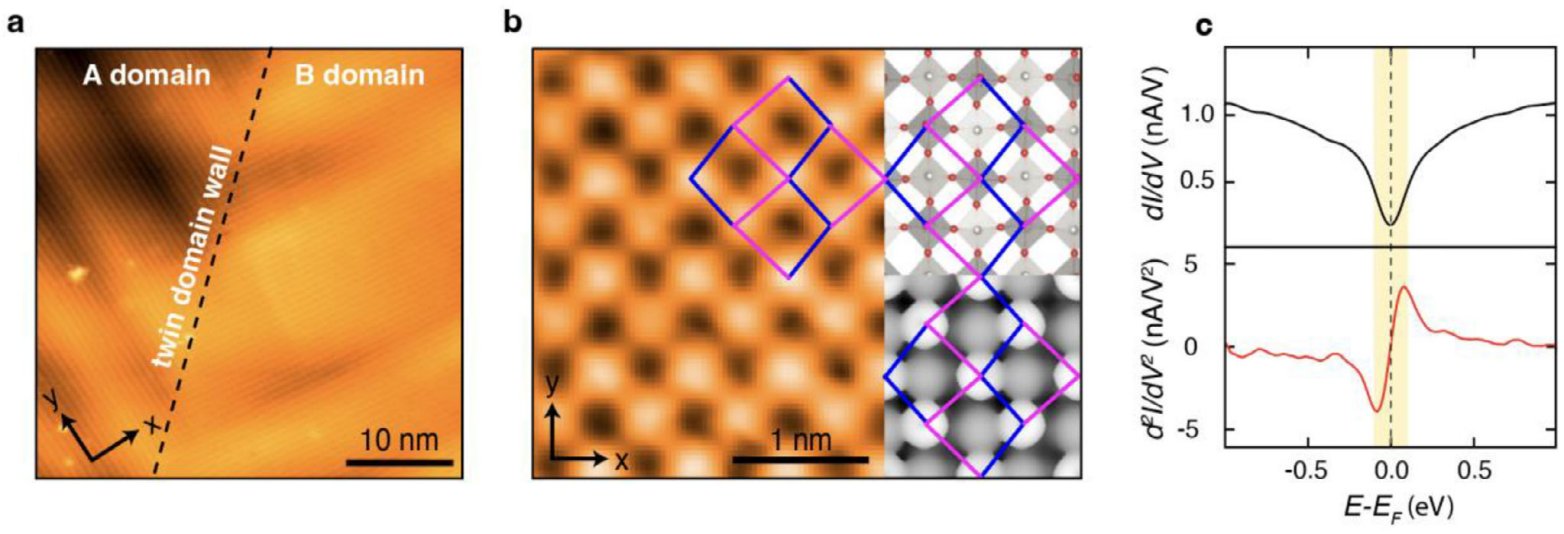
STM/STS analysis of a cleaved single‐crystalline WO_3_ film. a) STM image showing two distinct fine‐domains labeled as A and B with a macro‐domain boundary delineated by a dashed line (*V*_b_ = 3.0 V, *I*_t_ = 50 pA, scale bar 10 nm). b) (left) Atom‐resolved STM image of the *c*(2 × 2) surface reconstruction (*V*_b_ = 3.0 V, *I*_t_ = 15 pA, scale bar 1 nm). (top right) Atomic model of the cleaved WO_3_ surface. Here, white and red spheres represent tungsten and oxygen atoms, respectively, while the dark grey, light grey, and white areas denote octahedra, square pyramids, and voids, respectively. (bottom right) Corresponding simulated STM image obtained at *V*_b_ = 3.0 V (see Methods/DFT calculations for details). On (b), the *c*(2 × 2) motifs are superimposed, where blue and purple lines indicate shorter and longer nearest‐neighbor distances between top oxygen atoms. c) (top) Spatially averaged STS differential conductance (*dI/dV*) obtained at 88 K. (bottom) Corresponding numerical derivative (*dI* ^2^/*dV* ^2^) showing phonon‐assisted inelastic electron tunneling. The prominent inelastic phonon excitation of ≈±90 meV occurs around the Fermi level (highlighted by the yellow shaded region).

STM measurements reveal an atomically flat and clean WO_3_ surface (**Figure** **2**a). Similar to pristine WO_3_ films, both macro‐domains and fine‐domains coexist. High‐resolution STM images clearly show a robust and long‐range surface reconstruction (Figure 2b left), identified as the *c*(2 × 2) surface reconstruction with respect to the pseudocubic lattice of WO_3_ (Figure 2b top right). This reconstruction is attributed to the alternate removal of surface‐bound oxygen atoms, which agrees with previous literature^[^ ^33^, ^34^, ^35^ ^]^ (see Figure S2, Supporting Information for details). Our STM simulation (Figure 2b, bottom right) further confirms that the bright protrusions correspond to the intact surface oxygen atoms, while the dark depressions represent the oxygen vacant sites. This *c*(2 × 2) reconstruction is calculated to be the most stable among all possible reconstructions with the same oxygen deficiency (see Figure S5, Supporting Information for details).

To better understand the electronic properties of the surface reconstruction, we have performed STS measurements at 88 K. In sharp contrast to the insulating pristine WO_3_, the cleaved surface of WO_3_ exhibits a non‐vanishing density of states (DOS) at the Fermi level (Figure 2c top), suggesting the formation of a confined metallic surface state, likely due to the induced oxygen vacancies. Furthermore, the STS measurements reveal significant DOS enhancement at |*E*| > 100 meV, while the DOS at the Fermi level is suppressed. This gap‐like spectral feature in the tunneling spectrum likely originates from phonon‐assisted inelastic tunneling processes.^[^ ^44^, ^45^, ^46^, ^47^ ^]^ Phonon excitations, induced by tunneling electrons, appear as steps in *dI*/*dV* and as peak‐dip pairs in *d* ^2^ *I*/*dV* ^2^ at the corresponding phonon energies. Indeed, we observe this characteristic spectral feature in the numerical derivative of the STS spectrum (Figure 2c bottom). The clear peak‐dip pair at ≈±90 meV near the Fermi level indicates phonon‐assisted inelastic tunneling. This phonon mode aligns with the W‐O stretching vibrations reported in Raman spectroscopy.^[^ ^31^, ^32^, ^48^ ^]^ This finding will also be corroborated by ARPES measurements.

### 2.3 Electronic Structure and Polarons of WO_3_ Film After UV Photon Irradiation

**Figure 3:**
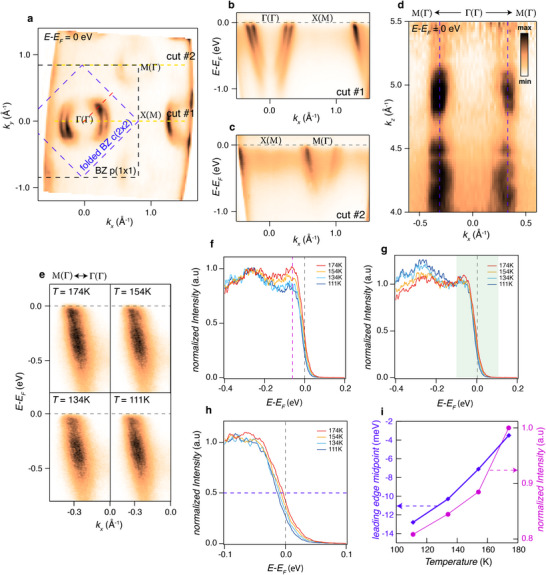
Electronic structure and polarons of WO_3_ film after UV photon illumination. a) Fermi surface of WO_3_ measured at a temperature 200 K with a photon energy of 58 eV. The black and blue dashed rectangles represent the *p*(1 × 1)Brillouin zone and reduced Brillouin zone by *c*(2 × 2) surface reconstruction, respectively. The high symmetry points of each Brillouin zone are separately indicated. b,c) Band dispersions along high symmetry lines indicated with yellow dashed lines in (a). d) k_z_ dependence of the electronic structure of WO_3_. e) Temperature dependence of the electronic structure of WO_3_ along red dashed line in a, taken at photon energy of 85 eV. f,g) Temperature‐dependent energy dispersion curve (EDC) stacks, normalized by intensity at the hump region (*E‐E_F_* = −270 meV) f) and by intensity at the peak region (*E‐E_F_* = −65 meV) g). h) A zoom‐in version of (g). i) Temperature dependence of leading‐edge midpoint shift (left) and that of the integrated intensity of peak (right). The midpoint is indicated with a blue dashed line in (h) and the peak position is indicated in a purple dashed line in (f).

Including possible polaronic behavior, further investigation of the induced metallic surface state has been carried out with ARPES. In **Figure** **3**a, the overall band structure of the metallic states is summarized. The signature of surface reconstruction is also captured with the periodicity of the electronic structure. Fermi surface exhibits a folded band at the M point of the *p*(1 × 1) Brillouin zone (BZ), originating from the Γ point of the *p*(1 × 1) BZ. This observation is consistent with the *c*(2 × 2) reconstruction revealed in our STM measurement. As a result, two concentric circular Fermi surfaces repeatedly appear at the center of the *c*(2 × 2) BZ, depicted by the blue dashed line, rather than the periodicity of the *p*(1 × 1) BZ (black dashed line). This *c*(2 × 2) reconstruction is also confirmed at the high binding energy region (Figure S4, Supporting Information). Band dispersions along the Γ−Μ high symmetry lines of the 1st and 2nd BZ, shown in Figure 3b,c (cut#1 and #2 in Figure 3a), exhibit the two concentric electron band dispersions, which are identical at both cuts. These bands are barely dispersive along the *k_z_* direction (Figure 3d), indicating the 2D confined nature of the state. The 2D nature and periodicity of the electronic structure led us to conclude that 2DEG states are confined to the cleaved surface of the WO_3_ film. In addition, the size of the Fermi surface with respect to the BZ size yields a high carrier density of 2.6 × 10^14^ cm^−2^.

Notably, the spectral weight at binding energies below 0.5 eV is apparently suppressed, which is often attributed to strong electron‐electron or electron‐phonon coupling in correlated systems.^[^ ^28^ ^]^ In addition to that, the suppression of spectral weight at ≈90 meV appears persistently across a wide temperature range (see Figure 3e). Such suppression renders a famous peak‐dip‐hump feature^[^ ^7^, ^20^, ^21^ ^]^ in the spectrum (Figure 3f), which was previously attributed to the spectral function of non‐interacting polarons, consisting of a low‐energy quasi‐particle peak representing polaron motion and a high‐energy incoherent resonance. Also, the presence of dip can be understood as energy‐selective suppression of spectral weight observed for the small polaron system; the repeated suppression of spectral weight along the band dispersion was captured, where the energy positions of suppression correspond to the integer multiple of the participating phonon energy scale.^[^ ^24^ ^]^ Indeed, the characteristic energy scale of 90 meV corresponds to the optical phonon mode of WO_3_,^[^ ^31^, ^32^, ^48^ ^]^ further supporting the polaronic nature of the spectrum. Finally, the spectral weight at the Fermi level diminishes as the system is cooled (Figure 3i right), which is in parallel with the observation from the phonon‐assisted tunneling (Figure 2c). This suppression of low‐energy spectral weight is attributable to the loss of coherence in polaronic motion, possibly due to strong carrier‐lattice interactions. Interestingly, the energy window of the suppression is not only limited near the peak but also reaches the Fermi level. To further characterize it, the spectra were normalized with peak intensity (Figure 3g), which reveals the shift in the leading edge of the spectrum (Figure 3h,i left).

### 2.4 Calculated Electronic Structure and Polaron States of WO_3_ Film

**Figure 4:**
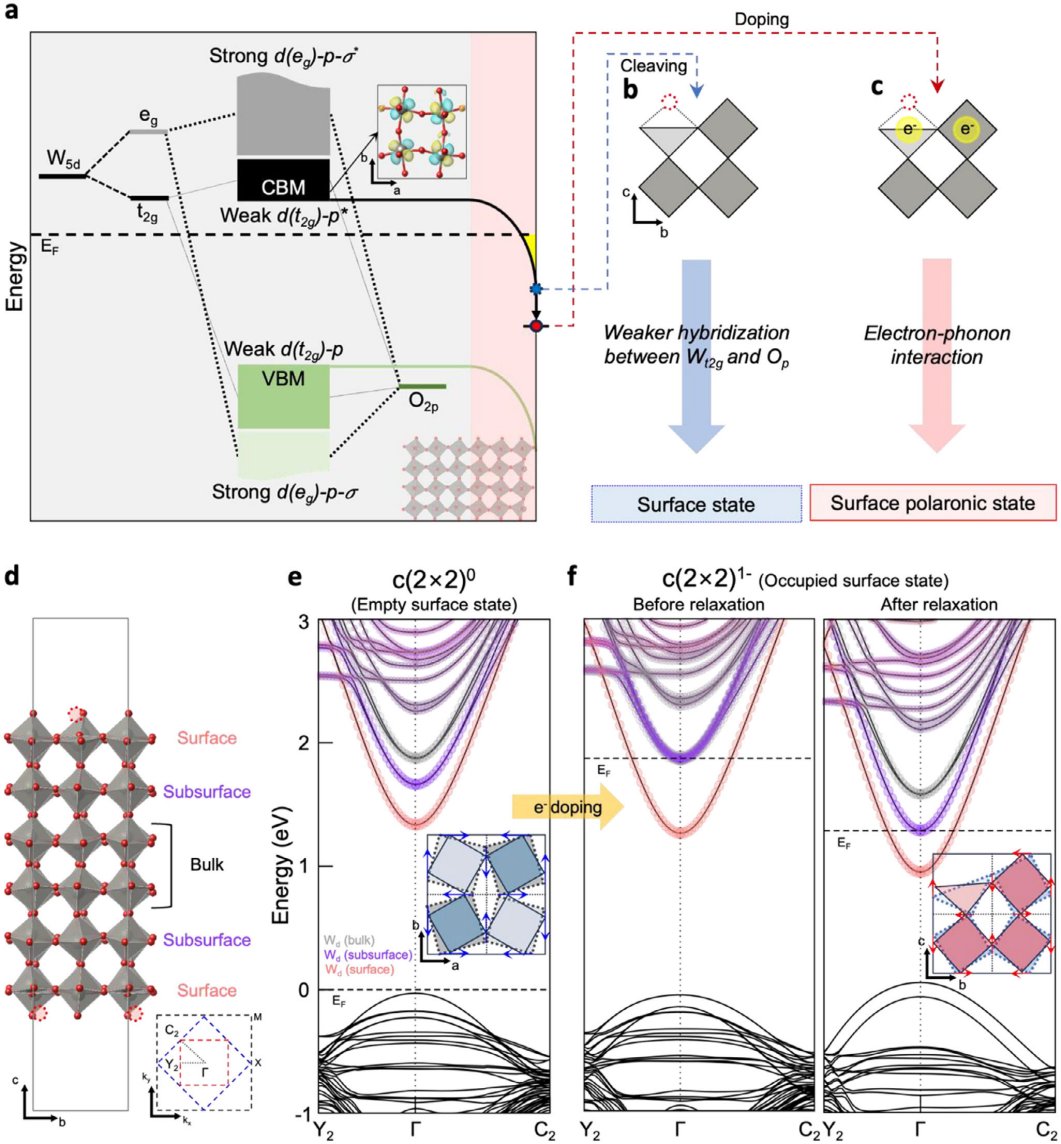
Calculated electronic structure and polaron states of WO_3_ film. a–c) Schematic illustration of the bulk's electronic structure with surface band bending (encompassing wavefunction plot at the Γ point of the conduction band minimum (CBM)), an unoccupied surface state before electron capture, and an electron polaronic surface state after electron capture. The positive and negative phases of the wavefunction are denoted in yellow and light blue, respectively. Blue‐dotted circle and red solid circle represent the surface state and the polaronic state, respectively. Red‐dotted circles represent oxygen vacancies. d) Side view (left) and the projected 2D Brillouin zone (bottom right) of the symmetric *c*(2 × 2) surface model. A (1 × 1 × 3) supercell is applied, with atomic positions within the bulk region kept constant. Surface and subsurface atoms are in antiphase rotation with each other. Red‐dotted circles depict oxygen vacancies of the front octahedra (Figure S5, Supporting Information for details). Black and blue dashed lines correspond to the 2D Brillouin zone of Figure 3a, and the red dashed line represents that of the surface model. e,f) Fat band structures of each layer's W‐5*d*(*t_2g_*) before/after electron capture. The surface, subsurface, and bulk states are represented in orange, purple, and grey shades, respectively. Insets exhibit the rotational alterations of square pyramids and octahedra on the surface. The zero energy corresponds to the valence band maximum (VBM) of the band structure prior to electron capture.

Now, we are ready to systematically investigate the microscopic nature responsible for the observed polaronic state on the WO_3_ surface through DFT calculations. The methodology entailed the following steps: i) identifying the character of the bulk conduction band minimum (CBM) to comprehend the foundational origin of the surface state (**Figure** **4**a), ii) analyzing the *c*(2 × 2) surface reconstruction without adding electrons (Figure 4b), and iii) examining the stabilization of the surface state with electrons, leading to polaron formation (Figure 4c).

To identify the microscopic origin of the bulk CBM, we first consider the splitting of the five W‐5*d* orbitals in the cubic octahedron into *e_g_* and *t_2g_* orbitals. Once octahedral rotation/tilting sets in, in the monoclinic P2_1_/n phase of WO_3_, the hybridization between W*t_2g_* and O*p* orbitals is allowed and yields CBM as antibonding state of W*t_2g_*‐O*p* hybridization (Figure S6b, Supporting Information). Note that the rotation also causes an upward shift in the CBM compared to the cubic phase.^[^ ^36^ ^]^ Next, we construct a symmetric and stoichiometric (1 × 1 × 3) slab model with oxygen vacancies at both surfaces, as depicted in Figure 4d (see Figure S7, Supporting Information for details on the dependency of slab size effect). The calculated atomic structures show that both surface and subsurface atoms tend to diminish the *ab*‐plane octahedral rotation, converging toward a symmetric cubic phase (inset of Figure 4e). The surface octahedra exhibit a rotational restoration of ≈6 degrees, while the subsurface octahedra restore by 1–3 degrees (Figure S8, Supporting Information), resulting in a downward shift of the CBM in each layer sequentially; the surface (orange) and subsurface (purple) states are shifted by 0.55 and 0.2 eV with respect to the bulk CBM (grey), as shown in Figure 4e, respectively.

The stoichiometric *c*(2 × 2) surface model, which has two surfaces symmetrically above and below, is charge neutral without any doping capability, thus exhibiting semiconducting character with an appreciable bandgap (Figure 4e). However, because the cleaved WO_3_ surface in the experiment is heavily *n*‐doped, we introduce one extra electron per *c*(2 × 2) surface unit cell to occupy both the empty surface and subsurface states (Figure 4f, the left panel). The doped electrons in the surface states exhibit localization, triggering the relaxation of the surrounding atoms (Figure S9, Supporting Information for details). In this case, both the surface and subsurface atoms restore the *bc*‐plane octahedral tilting by 2–3 degrees toward the cubic structure, further diminishing the W*t_2g_*‐O*p* hybridization (inset of Figure 4f; Figure S8, Supporting Information for details). This leads to a further lowering of the surface and subsurface states by 0.3–0.6 eV, as shown in Figure 4f from left to right panel.

This mechanism of the downward shift of surface/subsurface states, associated with the relaxation of octahedral distortion, is indicative of polaron formation. Indeed, our calculation is in parallel with recent discussions on the formation of antidistortive polarons in WO_3_, which locally compensates the intrinsic lattice distortions of the system.^[^ ^36^ ^]^ Additionally, it is noteworthy to note at this point that electrons are localized in all topmost W atom sites of a *c*(2 × 2) structure (Figure S9, Supporting Information). This observation implies the formed polarons exhibit an ordered configuration with the *c*(2 × 2) symmetry. Moreover, such polaron ordering may also be associated with the arrangement of bipolarons, i.e., pairs of bound polarons, that are localized across two adjacent *c*(2 × 2) unit cells. We note that this ordering should be distinguished from the Jahn‐Teller polaron, since the stabilization of the surface/subsurface state does not involve the lifting of orbital degeneracy. Nevertheless, this ordered nature of these polarons is reflected by the robust polaronic signature, even at the relatively high electron carrier density, which typically induces polaron collapse, as seen in SrTiO_3_ with densities higher than 1.2 × 10^14^ cm^−2^. Indeed, our observations parallel those in manganites, where scattering experiments have revealed a polaron lattice and the peak‐dip‐hump feature in the photoemission spectra of the metallic bulk electronic structure against high carrier density.

## 3 Conclusion

This unique ordered polaronic state, stabilized at high electron densities and tunable via external stimuli, opens up unprecedented opportunities to explore correlated polaronic states and/or collective motion of polarons, and exotic electronic phases dressed by them. One immediate and prime subject of interest is the role of polarons in potential superconductivity with critical temperatures (*T*_C_) exceeding 80 K in WO_3_.^[^ ^42^ ^]^ Indirect observations in several reports suggest that microstructural deformations result in superconductivity on the surface of Na‐doped WO_3_, and with doping of other alkali metals such as Li, Cs, H, etc.^[^ ^37^, ^38^, ^39^, ^40^ ^]^ Furthermore, this exotic conduction phenomenon has also been measured in pure WO_3_, where twin walls or oxygen deficiencies induce similar structural deformations.^[^ ^41^, ^42^, ^43^ ^]^ With the insights gained from the present study, we can revisit this intriguing phenomenon in light of polaron ordering, prompting further experimental and theoretical exploration. Likewise, this platform allows for the exploration of a richer variety of matter phases, such as tracing the evolution of pseudogaps across the melting of polaron order. The nature of polaronic state as a surface 2DEG adds further merit, offering more tuning parameters for manipulating the polaronic state, thereby enhancing the versatility of the system.

## 4 Experimental Section

### Epitaxial Film Growth

The single crystal WO_3_ film with ≈2 µm thickness was grown on YAlO_3_ (110)_o_ substrate by pulsed laser deposition,^[^ ^49^ ^]^ using the coherent COMPex PRO 205F KrF excimer laser with *λ* = 248 nm. The sintered WO_3_ target was used for WO_3_ film deposition. Before deposition, the YAlO_3_ substrate was annealed at 1000 °C for 2 h to clean the surface of the substrate. During the film deposition, the growth temperature was set to be 800 °C and the distance between the target and substrate was ≈4.7 cm. An oxygen partial pressure of 70 mTorr, laser fluence of 0.8 J cm^−2^ and repetition rate of 10 Hz were used. The thickness was estimated by growth time and macrodomain widths according to the investigation of scaling laws.^[^ ^49^ ^]^ After deposition, the sample was cooled to room temperature at a rate of −10 °C min^−1^ in an oxygen environment of 500 Torr to remove oxygen vacancies in the pristine film. Surface morphology of the WO_3_ film was characterized using a commercial atomic force microscope (MFP‐3D Infinity, Asylum Research) in the contact mode. A common silicon cantilever (HQ:NSC35, MikroMasch) was used for the surface topography measurement.

### STM/STS Measurements

To ensure an atomically clean surface, the WO_3_/YAlO_3_ sample was cleaved in an UHV environment (<1 × 10^−10^ Torr) following a cooling period of 30 min at temperatures below 150 K (see Figure S1, Supporting Information, for details). Then, the sample was immediately transferred to the UHV STM chamber, which was maintained at a temperature of 88 K. For the STM/STS measurements, electrochemically etched tungsten (W) tips were employed. Prior to their use in the experiment, the metallic state of these STM tips was verified and/or prepared on a clean Cu(100) surface. All STM images were obtained in constant current mode. Differential tunneling conductance spectra were recorded with the STM feedback loop disabled, using a lock‐in amplifier. A modulation voltage of 10 mV at 971 Hz was applied during lock‐in detection.

### ARPES Measurement

The ARPES measurements were performed at beamline 4.0.3 (MERLIN), Advanced Light Source, Lawrence Berkeley National Laboratory. Samples were cleaved under a pressure below 5 × 10^−11^ Torr at 15 K, and the measurements were carried out under the same conditions. Synchrotron photon irradiation was carried out by exposing the cleaved sample to high flux UV with a kinetic energy of 90 eV for an hour (Figure S3, Supporting Information).

### DFT Calculations

DFT calculations were conducted using the Vienna ab initio Simulation Package (VASP) in conjunction with projector‐augmented wave (PAW) pseudopotentials sourced from the VASP database.^[^ ^50^, ^51^ ^]^ To reproduce the distortion of WO₆ octahedra, which is important for explaining polarons, the regularized‐restored strongly constrained and appropriately normed (r^2^SCAN) functional within the meta‐generalized gradient approximation (meta‐GGA) was chosen.^[^ ^52^ ^]^ The DFT + U method does not fully capture this distortion, while the hybrid functional comes with a high computational cost. Moreover, polaron formation for the hybrid functional shows no significant difference compared to that for the r^2^SCAN functional. We performed spin‐unpolarized calculations, as the conduction band in WO_3_ is highly dispersive (Figure 4e). This is justified by the Stoner criterion, which indicates that the exchange interaction and the DOS at the Fermi level are insufficient to induce spontaneous spin polarization. A plane‐wave basis energy cutoff was used of 600 eV. For the bulk framework, a (2 × 2 × 2) mesh was used for *k*‐point sampling, whereas for the slab, a (2 × 2 × 1) mesh was applied. The symmetric slab, as illustrated in Figure 4d, comprised a 1 × 1 × 3 supercell of the unit cell, separated by a 15 Å vacuum. Atomic positions in the middle layer were fixed. All‐atom positions were relaxed until the Hellman‐Feynman force exerted on each atom was less than 0.01 eV Å^−1^. The calculated bandgap for monoclinic WO_3_ (1.98 eV) was found to be smaller than the experimentally determined bandgap range of 2.60–3.25 eV.^[^ ^53^, ^54^, ^55^ ^]^ However, the optimized lattice constants (*a* = 7.376 Å, *b* = 7.576 Å, *c* = 7.681 Å) show a qualitative concordance with the experimental values (*a* = 7.303 Å, *b* = 7.538 Å, *c* = 7.692 Å).^[^ ^56^ ^]^ The STM simulation was conducted in the constant current mode with the Tersoff‐Hamann approach^[^ ^57^, ^58^ ^]^ for the electronic states from 2.3 to 5.3 eV in Figure 4e of the charge neutral *c*(2 × 2) surface model, which may correspond to the experimental window from the Fermi energy and the bias voltage of 3 V. A (6 × 6 × 1) mesh of *k*‐points was used to obtain the electronic structure of the slab for the STM simulation.

## Conflict of Interest

The authors declare no conflict of interest.

## Supporting information



Supporting Information

## Data Availability

The data that support the findings of this study are available from the corresponding author upon reasonable request.
