# Supplementary material for: Unscreened Polaron Ordering Against High Electron Density on Mechanically Cleaved Surfaces of WO3 (001) Films
Source: Small. 2025 Sep 1;21(39):e04162. doi: 10.1002/smll.202504162 (PMC12490174; doi:10.1002/smll.202504162)
Supplement: Supplementary file 1 — Supporting Information [file SMLL-21-e04162-s001.docx]

Supporting Information

**­­­Unscreened polaron ordering against high electron density on mechanically cleaved surfaces of WO_3_ (001) films**

Gyubin Lee^1,†^, Jeongdae Seo^1,†^, Wooin Yang^2,†^, Ji-Ho Mun^1,†^, Minho Kang^1^, Ho-Hyun Nahm^1^, Yong-Hyun Kim^1,3,*^, Tae-Hwan Kim^2,*^, Chan-Ho Yang^1,*^, Yeongkwan Kim^1,*^

^1^Department of Physics, Korea Advanced Institute of Science and Technology, Daejeon 34141, Korea.

^2^Department of Physics, Pohang University of Science and Technology, Pohang 37673, Korea.

^3^School of Physics, Institute of Science, Suranaree University of Technology, Nakhon Ratchasima 30000, Thailand.

†These authors contributed equally to this work.

*E-mail: yeongkwan@kaist.ac.kr, chyang@kaist.ac.kr, taehwan@postech.ac.kr, yong.hyun.kim@kaist.ac.kr


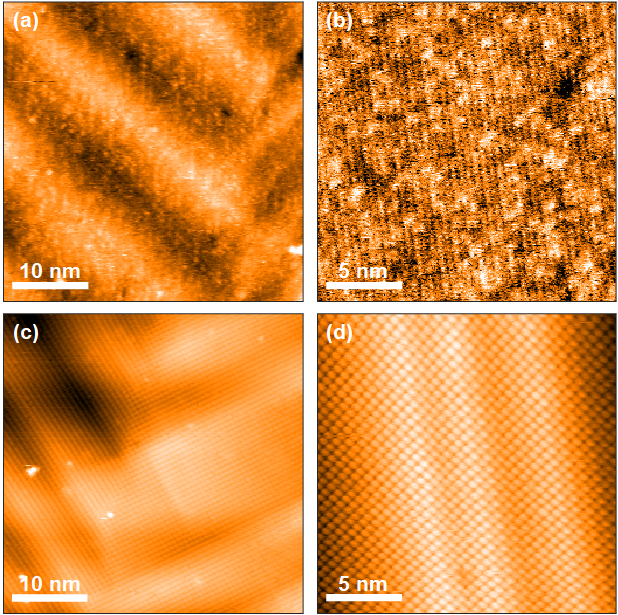


**Supplementary Figure 1 | Impact of cleaving temperature on surface morphology.** **a,b,** STM images obtained at room temperature, following a cleaving process also conducted at room temperature. **c,d,** STM images taken at 88 K, after cleaving at <150 K. Imaging parameters for the STM images are as follows: 1 V, 20 pA **(a)**; 1 V, 100 pA **(b);** 3 V, 50 pA **(c);** 1 V, 100 pA **(d)**.

Figure S1 provides a clear and comparative visualization of how cleaving temperature significantly influences the surface morphology of our sample. Figures S1a and S1b show STM images captured at room temperature after the sample was cleaved at the same temperature. They reveal certain characteristic features such as the presence of a twin domain structure. However, these images also indicate a lack of atomic homogeneity across the surface. In contrast, Figures S1c and S1d were taken at 88 K, but after the sample was cleaved at temperatures below 150 K. These images contrast markedly with those obtained from room-temperature cleaving. The most notable difference is the achievement of an atomically homogeneous surface, a feature that was not observed in the samples cleaved at room temperature. This suggests that lower cleaving temperatures facilitate a more uniform and orderly surface structure, possibly due to reduced thermal agitation at the time of cleaving.


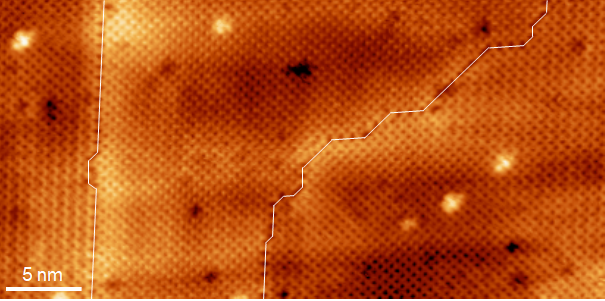


**Supplementary Figure 2 | STM image showing different** $\boldsymbol{c(2\times2)}$ **domains**. The domain boundaries are marked with white lines for clarity. Imaging conditions: 2 V, 30 pA.

Figure S2 presents a variety of $c\left( 2\times2 \right)$ domains observed on the cleaved surface, indicating that the $c\left( 2\times2 \right)$ reconstruction likely occurs subsequent to the cleaving process. Initially, when the surface is cleaved, oxygen vacancies (O-vacancies) are expected to be randomly distributed across it. Following the cleaving, these O-vacancies begin to reorganize into the more energetically stable $c\left( 2\times2 \right)$ structure. This reorganization process involves the random initiation of $c\left( 2\times2 \right)$ domain nucleation across different areas of the surface. As these domains develop, they eventually converge, forming the distinct domain boundaries that are evident in Figure S2. This pattern of domain formation and boundary creation provides insight into the dynamic process of surface reconstruction following mechanical cleaving.


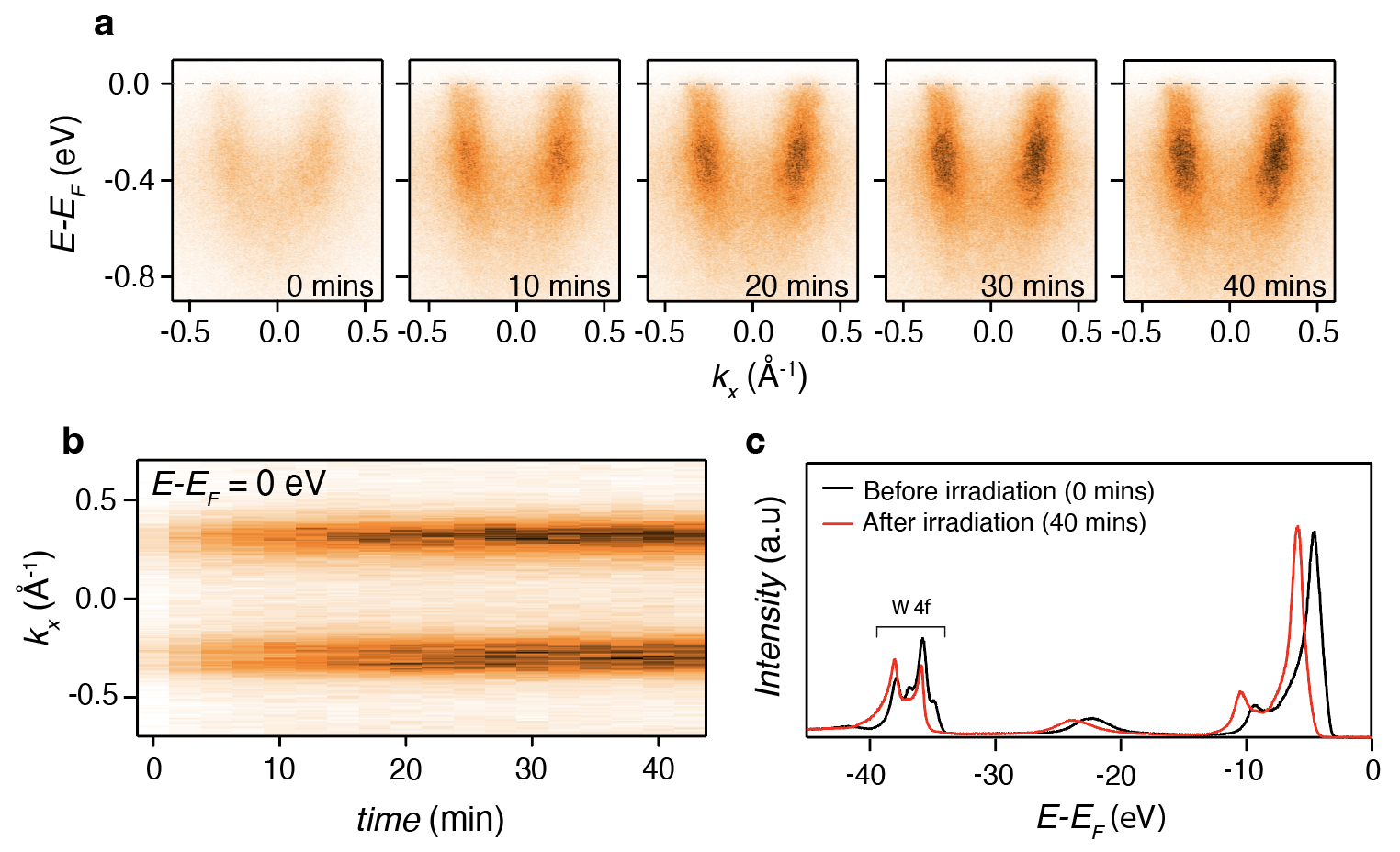


**Supplementary Figure 3 | Effect of UV irradiation on WO_3_ (001) surface. a,b,** Time-dependent change of ARPES spectral weight of WO_3_ at Γ(Γ) near the Fermi level under UV irradiation. **c,** Comparison of core level spectra of WO_3_ before and after UV irradiation.


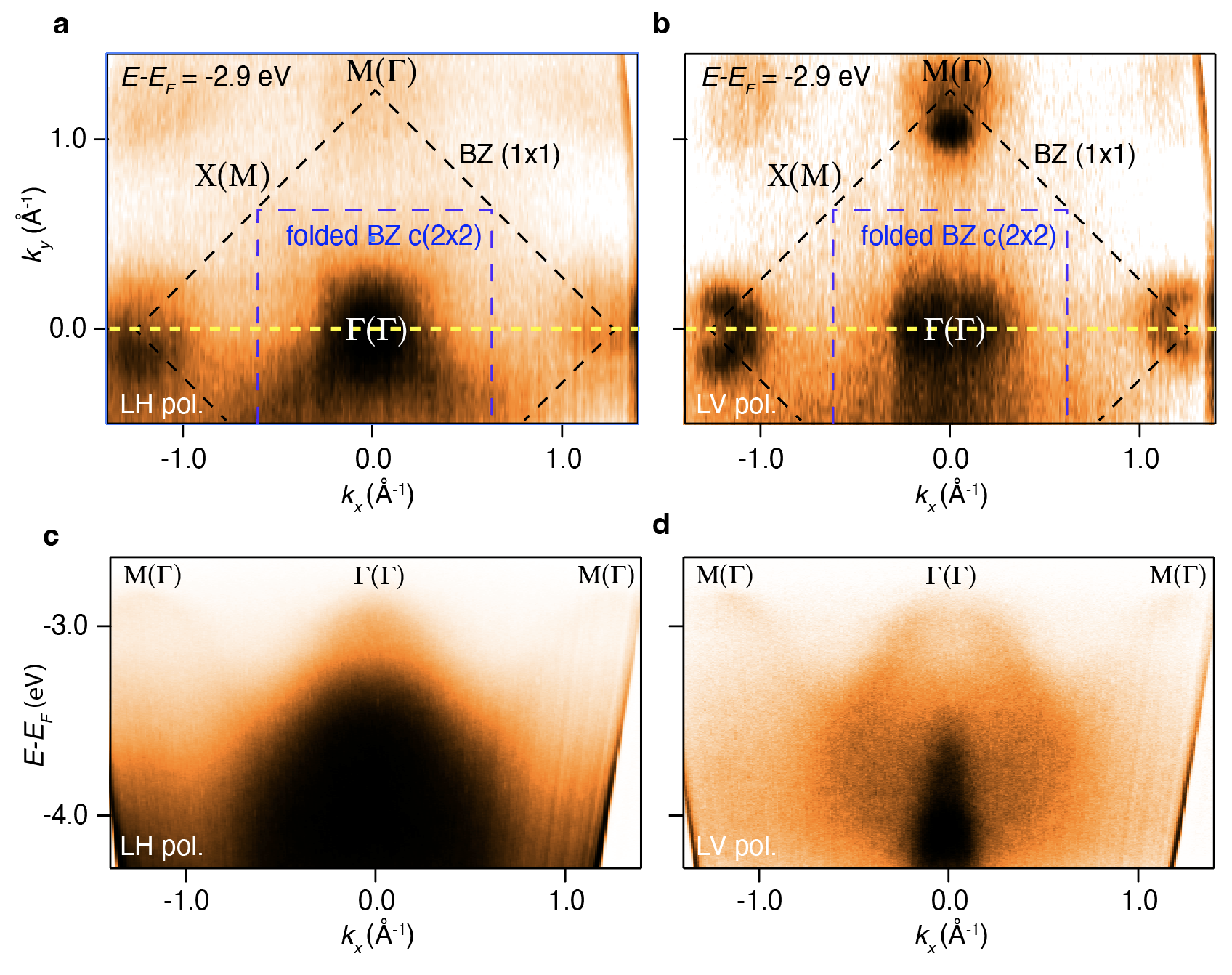


**Supplementary Figure 4 | Measured high-binding electronic structure of WO_3_ a,b,** Constant energy map of WO_3_ at binding energy 2.9 eV measured with (**a**) LH-polarized and (**b**) LV-polarized photons. **c,d,** Dispersions along high symmetry lines indicated in (**a**) and (**b**) (yellow dashed lines), respectively.


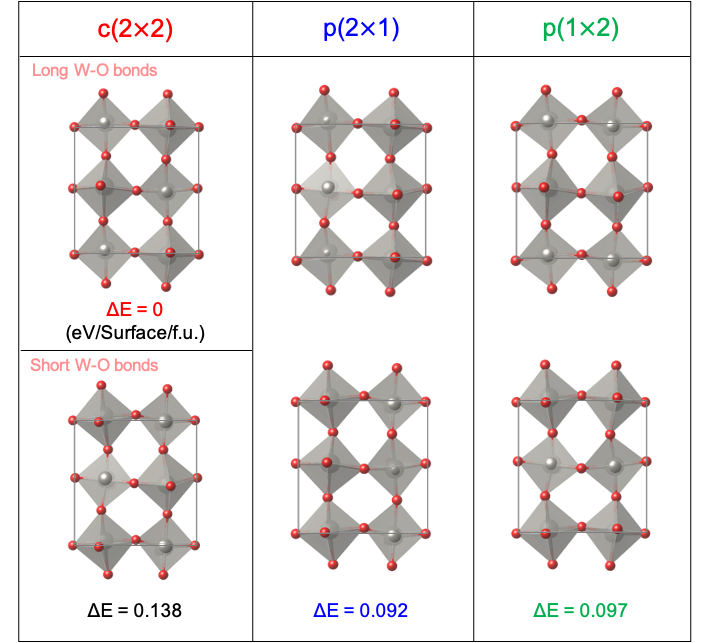


**Supplementary Figure 5 | Comparison of surface energy for all possible patterns (i.e., top view of** $\boldsymbol{c(2\times2)}$**,** $\boldsymbol{p(2\times1)}$**, and** $\boldsymbol{p(1\times2)}$**).** The difference of surface energy was calculated based on the structure on the left above.

As we mentioned in the main text, our scanning tunneling microscopy (STM) experiments revealed a predominant $c(2\times2)$ surface reconstruction due to the alternate removal of two surface-bound oxygen atoms. Our DFT calculations further corroborated the $c(2\times2)$ configuration's significant energetic stability (0.138 eV/surface/f.u.)—the outcome of eliminating two uppermost oxygen atoms possessing the longest W-O bonds in the quartet—compared to an identical $c(2\times2)$ structure formed by the removal of two oxygen atoms with shorter W-O bonds.


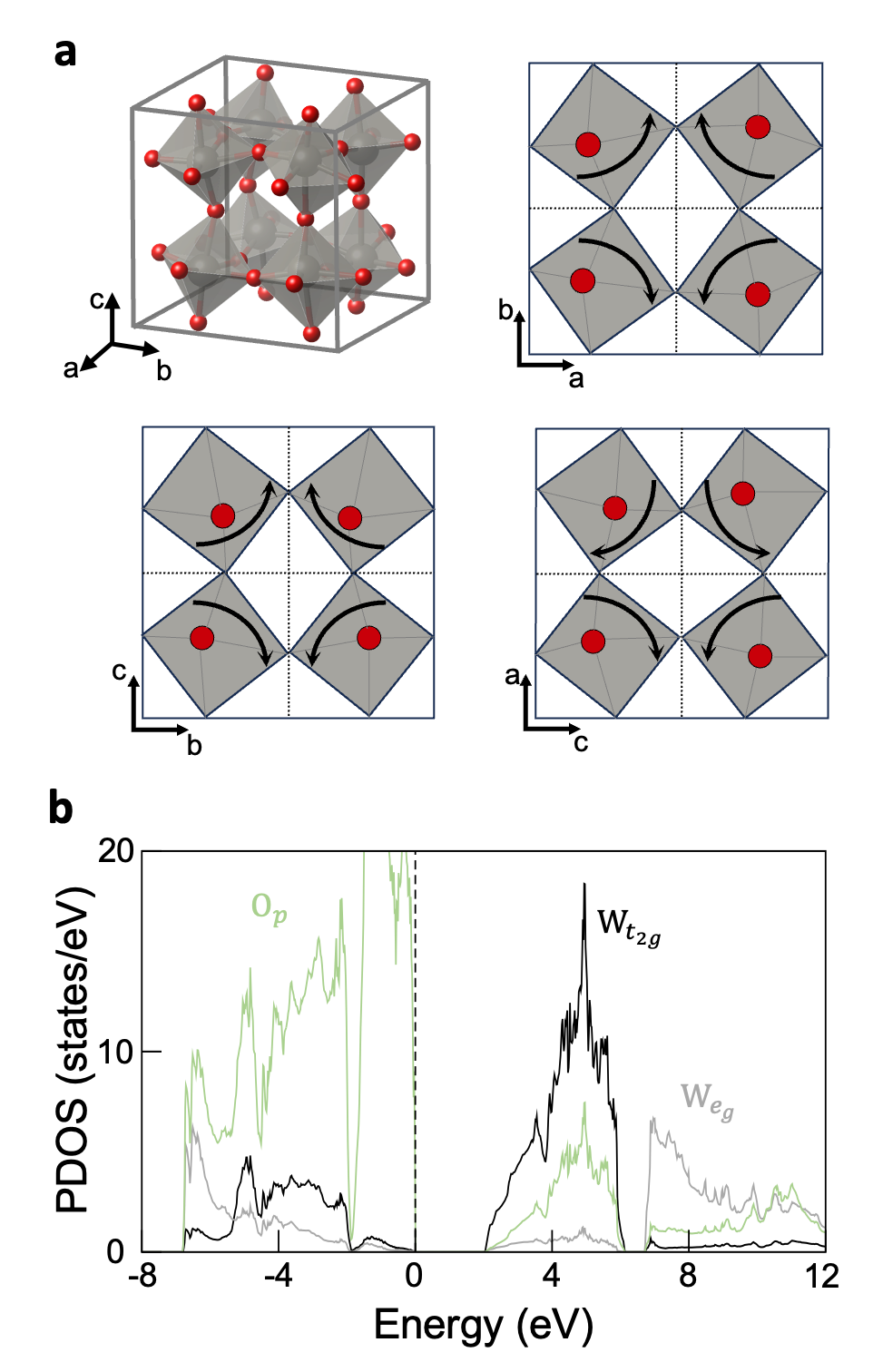


**Supplementary Figure 6 | Atomic and electronic structures of bulk WO_3_. a,** Atomic structures of bulk WO_3_. Black arrows show the cooperative rotation of the octahedra. **b,** Projected density of states (PDOS) of bulk WO_3_. The black, grey, and green lines represent W-5d(t_2g_), W-5d(e_g_), and O-2p states, respectively. The Fermi energy is set to 0 eV at the valence band maximum (VBM).


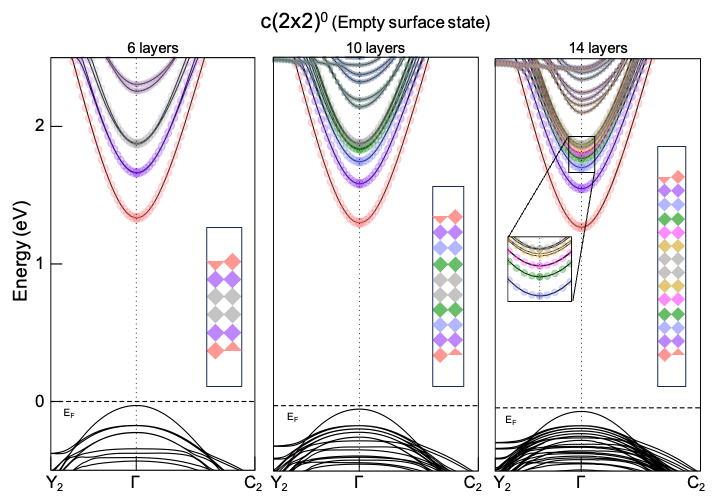


**Supplementary Figure 7 | Fat band structures of W-5d(t_2g_) of the slab model according to the number of layers.** Insets in the band structures schematically depict respective layers with corresponding colors on the side view of the slab model. Atomic positions with grey octahedra are fixed. The zero energy is aligned on the VBM with a 6-layer structure.


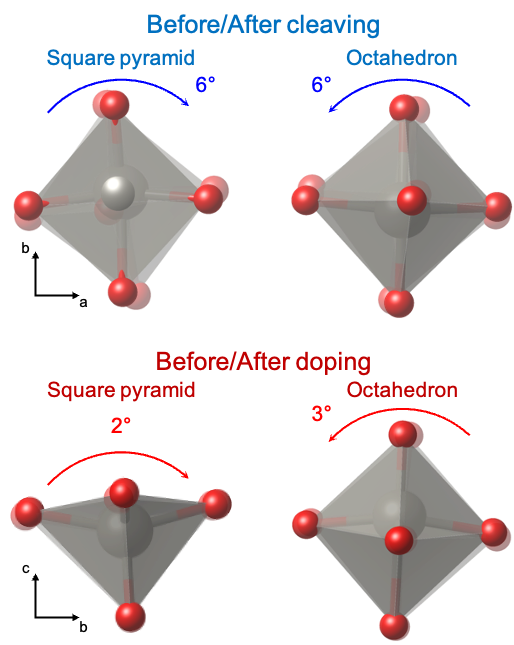


**Supplementary Figure 8 | Rotational variation in the atomic structure of square pyramids and octahedra for the** $\boldsymbol{c(2\times2)}$ **surface before (transparent) and after (opaque) cleaving/doping.** Blue and red arrows depict the rotational direction.


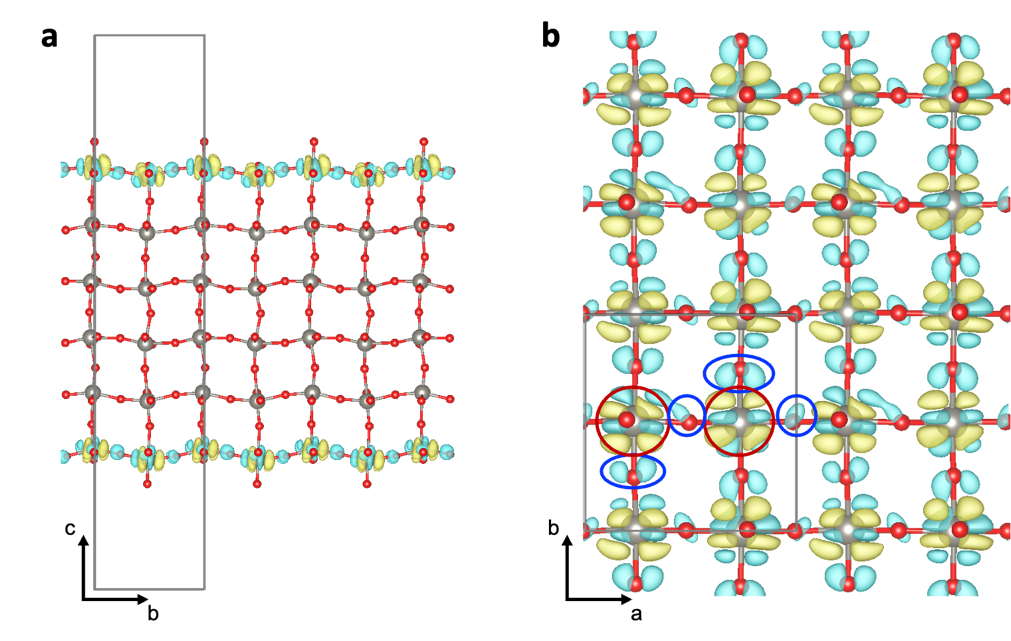


**Supplementary Figure 9 | Charge density difference between before and after atomic relaxation. a,** Side view of charge density difference. Light blue and yellow isosurfaces show the negative and positive values, respectively. The isosurface level is set to be $1.1\times{10}^{-11}$. **b,** Top view of charge density difference. Red and blue circles represent electron localization on W atoms and removal of charge density on O atoms, respectively.
